# Development and optimization of sustained release triptolide microspheres

**DOI:** 10.1371/journal.pone.0292861

**Published:** 2023-10-19

**Authors:** Hui-lin Zeng, Qian Qiu, Ting-xiong Fu, Ai-ping Deng, Xiang-yang Xie

**Affiliations:** 1 Department of Pharmacy, Central Hospital of Wuhan, Tongji Medical College, Huazhong University of Science and Technology, Wuhan, Hubei, China; 2 Department of Pharmacy, General Hospital of Central Theater of the PLA, Wuhan, Hubei, China; Addis Ababa University College of Health Sciences, ETHIOPIA

## Abstract

Rheumatoid arthritis is considered a chronic systemic autoimmune disorder that may cause joint destruction. Triptolide, an active component isolated from *Tripterygium wilfordii* Hook.f., is considered to have promising potential for clinical use in treating rheumatoid arthritis. However, its clinical application has been limited by the narrow therapeutic window, side effects associated with plasma drug fluctuations, low oral bioavailability, and poor patient compliance with the long and frequent dosing regimen. An extended drug release preparation may address these limitations. The aim of this work was therefore to develop, formulate and optimize sustained release triptolide microspheres with poly (lactide-co-glycolide) (PLGA). Triptolide-loaded microspheres were prepared using PLGA as the matrix polymer, dichloromethane as the oil phase, and polyvinyl alcohol (PVA) as the matrix forming emulsifier. An oil-in-water (O/W) emulsion solvent evaporation technique was utilized to prepare the microspheres. Surface response methodology (RSM) coupled with central composite design (CCD) was used to optimize the formulation and a total of twenty formulations were prepared. PVA concentration (X_1_), PLGA concentration (X_2_), and theoretical drug content (X_3_) were selected as independent variables; and drug content (Y_1_), encapsulation efficiency (Y_2_), mean diameter (Y_3_) and the initial release during the first day (Y_4_) were taken as the response variables. The optimized formulation showed mean diameter of 42.36 μm, drug content of 7.96%, encapsulation efficiency of 80.16% and an initial release of 14.48%. The prepared microspheres exhibited a sustained release profile of triptolide *in vitro* over 4 weeks, which was wellfitted with a Korsmeyer-Peppas equation. However, the initial drug release (~14%) of triptolide-loaded microspheres was very high and should be specifically investigated in future studies. The results indicate that long-term sustained release microspheres of triptolide can be considered a strategy to overcome the low bioavailability and poor patient compliance with conventional triptolide tablets. The issue of initial burst release and *in vivo* evaluations should be specifically investigated in the future.

## Introduction

Rheumatoid arthritis (RA), associated with inflammatory manifestations in synovial joints and other organs (such as the heart and lungs), is considered a chronic systemic autoimmune disorder that may cause joint destruction [1]. Despite the ever increasing progress in medical science, the therapeutic needs for RA are still not well met. Epidemiological investigation revealed that the RA has a global prevalence of approximately 1% and varies highly with sex and race [2]. For RA patients, disease development involves progressive joint deformity, thereby leading to loss of function. In addition, extra-articular symptoms are also common in RA patients and affect important systems of the body, such as the heart (pericarditis), blood vessels (Raynaud’s phenomenon), lung (pulmonary nodules, pulmonary fibrosis), nerves (peripheral nerve entrapment, polyneuropathy) and eyes [3]. These disorders seriously affect the quality of life, longevity and work capacity of RA sufferers [4].

To date, there are many therapeutic agents available on the market to manage the symptoms of RA patients. Among these drugs, the Chinese traditional medicine (TCM) of *Tripterygium wilfordii* Hook.f. (TWHF) cannot be ignored for its favorable cost‒benefit ratio and special immunosuppressive efficacy in the treatment of RA [5]. TWHF has been used as an herbal remedy to treat arthritis and other autoimmune inflammatory disorders since the late Qing Dynasty (19^th^ century) in China [6], and it has been used as an antitumor agent in recent years [7]. Clinical trials in the United States have also confirmed the efficacy of TWHF in the treatment of RA [8, 9]. Currently, several TCMs made from TWHF are available on the market in China, including *Tripterygium wilfordii* tablets (approved in 1998), *Tripterygium* hypoglaucum hutch tablets (approved in 2002) and *Tripterygium wilfordii* glycoside tablets (approved in 1998) [10]. For patients with RA, these tablets are recommended for oral administration under the supervision of doctors.

**Fig 1:**
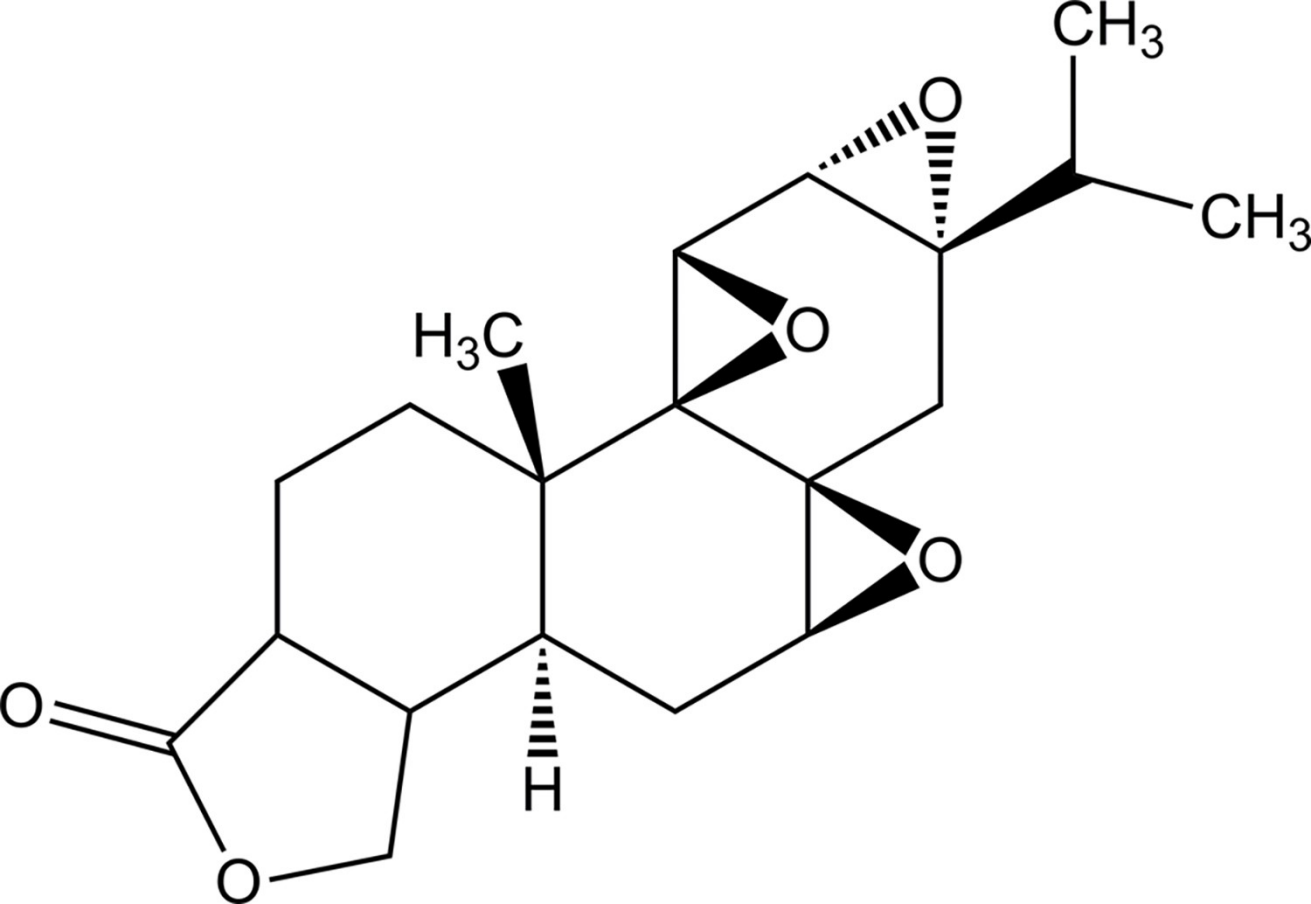
Chemical structure of triptolide: A bioactive diterpene triepoxide isolated from *Tripterygium wilfordii Hook F*.

Triptolide (Fig 1), a bioactive diterpene triepoxide isolated from TWHF, exhibits potent immunomodulatory, cartilage protective and anti-inflammatory activities [11], and it is considered to have promising potential to be used in the clinic to treat RA and other autoimmune diseases [7]. In the treatment of RA, extracts of TWHF (*Tripterygium wilfordii* tablets) are often recommended to be taken orally (3 times per day, 20–40 mg per time) over 4 weeks, in which the content of triptolide is 33 μg per tablet (20 mg of TWHF extract). For the treatment of inflammatory and autoimmune diseases, the dose doubled to 66 μg triptolide per time (3 times per day) was proven to be safe and effective [12, 13]. However, the clinical application of triptolide still faces several challenges, including multiorgan toxicity, a narrow therapeutic window [7], side effects associated with plasma drug fluctuations, low oral bioavailability due to its poor aqueous solubility [11], and poor patient compliance due to the long and frequent dosing regimen.

To address these limitations, a sustained release drug delivery system (DDS) for injection, which can deliver a relatively constant dose of triptolide for a long period of time, may provide an alternative to solve the aforementioned problems to some extent. Microspheres are innovative DDSs that possess favorable features and have been studied widely over three decades [14]. Microspheres can be classified into two types, biodegradable and nonbiodegradable, and biodegradable microspheres are preferable in practical applications. Biodegradable microspheres can extend the duration of drug action remarkably, reduce the dosing frequency and hence improve the compliance of patients. The total drug dose and adverse reactions related to waving blood drug levels can also be diminished due to the steady plasma concentration that the microsphere formulation will bring [15]. Furthermore, there is no need to implant biodegradable microspheres in surgical operations and remove them after drug release is completed. Moreover, by changing the route of drug administration from oral to depo injection, the bioavailability of triptolide can also be improved. However, microsphere preparations may face the problem of initial burst release, which is more prominent for narrow therapeutic index drugs such as triptolide. This issue should be addressed in the final phase of the dosage form study.

Poly (lactic-co-glycolic acid) (PLGA), an FDA approved biodegradable polymer for *in vivo* use, is one of the most widely used polymers for DDSs because of its biodegradability, biocompatibility and ease of processing [16]. Currently, there are several injectable microsphere products on the market using PLGA as their carrier (e.g. Lupron Depot^®^, Zoladex^®^, Risperdal Consta^®^) [17].

Taken together, the aim of the present study was to develop a sustained-release microsphere formulation of triptolide for the treatment of RA. Since there are few reports on the formulation of triptolide using sustained release microspheres, the objective of this study was therefore to develop, characterize, and optimize triptolide loaded microspheres to obtain a prolonged release system to treat RA. In this work an oil-in-water (O/W) emulsion solvent evaporation technique was utilized to prepare microspheres, and a response surface methodology (RSM) coupled with central composite design (CCD) was used to optimize the formulation. The size, morphology, encapsulation efficiency, and *in vitro* drug release feature of the optimized microspheres were also investigated.

## Materials and methods

### Ethics statement

This study did not involve nonhuman primates or other animals.

### Materials

Triptolide (99.7% purity) was purchased from Nanjing Zelang Pharmaceutical & Scientific Company (Jiangsu, China). Poly (D, L-lactide-*co*-glycolide) copolymers, PLGA (75:25) with molecular weights of 20 kDa, were supplied by the Jinan Research Institute of Medical Equipment (Shandong, China) in their capped forms (ester end group). Polyvinyl alcohol-124 (PVA) was obtained from the Beijing East Ring Union Chemical Plant (Beijing, China).

All other materials or solvents were of reagent or analytical grade.

### Experimental design

Before the application of formulation optimization, preliminary experiments were conducted to identify the critical formulation and process parameters. The single factor screening method [18, 19], also known as the one-factor-at-a-time (OFAT) method [20] was used in the preliminary trials. The levels of formulation variables were also determined in preliminary trials. The encapsulation efficiency (EE) of microspheres was selected as the response variable to evaluate formulation/process parameters during OFAT experiments. The OFAT studies provided the factors affecting EE and the setting of the levels for each factor. Factors that have marked effects on the EE of microspheres were identified, including the concentration of PVA (2–8%), PLGA concentration (100–400 mg·mL^-1^), and theoretical drug loading (1–12%). Other variables investigated in the preliminary experiments were the oil/water ratio (1:4–1:9) and stirring speed (200–1000 rpm).

Following the preliminary experiments, critical factors (PVA concentration, PLGA concentration, and theoretical drug loading) were selected to obtain the optimum formulation by a response surface methodology (RSM) using central composite design (CCD). The coded values of the factors and their levels (based on the results of preliminary experiments) are shown in Table 1. Design-Expert 8.0.5 software was applied to perform the RSM process and therefore to investigate the effects of variable factors on the system’s response [21, 22]. The experimental plan is shown in Table 2, where the actual order of the experimental arrangement is given randomly by the software. This means randomizing the order of runs from the fixed design (standard order Table 2). The number of replicates was 6 (standard order 15 to 20 in Table 2), and the number of independent experiments was 14 (standard order 1 to 14 in Table 2).

**Table 1:** Experimental levels of independent variables in central composite design.

Factor	Level (-1.732)	Level (-1)	Level (0)	Level (+1)	Level (+1.732)
X_1_/%	3.00	3.61	4.50	5.62	6.00
X_2_/ mg·mL^-1^	100	141	200	259	300
X_3_/%	4.00	5.62	8.00	10.38	12.00

**Table 2:** Experimental plan of central composite design.

Standard Order	X_1_/%	X_2_/ mg·mL^-1^	X_3_/%
1	3.61	141	5.62
2	5.39	141	5.62
3	3.61	259	5.62
4	5.39	259	5.62
5	3.61	141	10.38
6	5.39	141	10.38
7	3.61	259	10.38
8	5.39	259	10.38
9	3.00	200	8.00
10	6.00	200	8.00
11	4.50	100	8.00
12	4.50	300	8.00
13	4.50	200	4.00
14	4.50	200	12.00
15	4.50	200	8.00
16	4.50	200	8.00
17	4.50	200	8.00
18	4.50	200	8.00
19	4.50	200	8.00
20	4.50	200	8.00

The independent variables in CCD studies were PVA concentration (X_1_), PLGA concentration (X_2_), and theoretical drug content (X_3_). For each factor, the experimental range was selected based on the results of preliminary experiments. By using parameters in these selected ranges, microspheres could be prepared successfully with desired characteristics (drug content more than 2%, drug encapsulation efficiency more than 80%, mean diameter in the range of 10–60 μm). The dependent variables (responses) were drug content (Y_1_), encapsulation efficiency (Y_2_), mean diameter (Y_3_) and initial release (Y_4_, to calculate the cumulative percentage of the drug released in the first day after incubation).

In this study, Design-Expert 8.0.5 software was used to analyze the data of the optimization experiment (as shown in Table 3). Central composite design (CCD) was applied to investigate the effect of the three independent variables (factors) and their potential interactions. Twenty experiments were performed with three factors and five levels, and they were augmented with six replications at the central point to estimate the "pure error". The experimental data were input into the software to conduct multiple regression analysis.

**Table 3:** Microsphere formulation variables and physical properties (mean±SD).

Standard Order	Drug content (%)	Encapsulation efficiency (%)	Mean diameter (μm)	Initial release (%)
1	4.75	84.3	47.38	14.10
2	4.21	74.9	30.84	18.94
3	4.79	85.2	55.79	11.02
4	4.09	73.4	40.69	16.87
5	6.32	59.8	52.61	14.94
6	5.92	57.7	39.37	19.82
7	8.51	81.8	45.19	15.02
8	7.83	74.7	34.45	19.70
9	6.43	79.3	54.74	16.38
10	5.74	71.4	31.19	25.47
11	5.58	69.8	39.15	14.95
12	6.69	82.9	45.06	11.64
13	3.22	80.4	42.69	12.06
14	7.91	64.4	43.73	16.98
15	6.95	86.9	42.09	14.06
16	6.89	86.1	46.12	16.16
17	6.74	84.4	45.82	15.03
18	6.51	81.3	43.36	16.17
19	6.93	86.5	45.50	13.71
20	7.01	87.6	44.16	15.52
Range	3.22–8.51	57.7–87.6	30.84–55.79	11.02–25.47

### Preparation of microspheres

Triptolide loaded microspheres were prepared by a classic oil-in-water (O/W) emulsion solvent evaporation method [23]. In brief, PLGA (120 and 299.57 mg) and triptolide (theoretical drug content: 1.99, 3.21, 5.00, 6.80 and 8.02%, w/v) were dissolved in 10 mL of dichloromethane (DCM) as the oil phase. Then the mixed solution was dropped slowly into 100 mL of deionized water as an external phase containing varying amounts of PVA (concentration: 3.04, 3.63, 4.50, 5.37 and 5.96%, w/v) as an emulsifier and stirred by a propeller stirrer (SXJQ-1, Zhengzhou, China; 4-blade propeller stirrer with 20 mm stirrer diameter) at 800 rpm for 10 min at 25°C to produce an O/W single emulsion. The resulting emulsion was then poured into 200 mL of deionized water and stirred at 300 rpm on a magnetic stirrer (egg-shaped stirring bar with a length of 30 mm) for 4 h at 25°C to evaporate the DCM. The hardened microspheres were collected by filtering through filter paper and then rinsed three times with 10 mL of deionized water; after that, the microspheres were dried under vacuum at room temperature to obtain free flowing powder.

### Determination of triptolide content

The amount of triptolide contained in the microspheres was determined by an HPLC method. Briefly, 25 mg of triptolide loaded microspheres was dissolved in 5 mL of acetonitrile, diluted to 50 mL with methanol and vortexed for 1 min. The resulting solution was filtered through a 0.45 μm filter membrane and analyzed by an HPLC system (Agilent 1260, USA) with a reversed phase C_18_ column (5 μm, 4.6 mm × 250 mm, Elite Analytical Instruments Co., Ltd., China). The analysis temperature was 30°C and the detection wavelength set at 218 nm. The mobile phase consisted of water and methanol at a ratio of 60: 40. The flow rate was 1 mL/min and the injection volume was 20 μL. The related method validation data of this section are presented in S1 File.

The encapsulation efficiency (EE) and drug loading (DL) of the microspheres were calculated by the following equations [24]:

EE = (drug found in tested microspheres × microsphere weight of tested batch / tested microspheres weight / drug added in tested batch) × 100%

DL = (drug found in tested microspheres / tested microsphere weight) × 100%

Here, "drug found in tested microspheres" refers to the drug content detected by HPLC in the tested microspheres; "drug added in tested batch" refers to the theoretical drug added in the tested formulation batch. All measurements were performed in triplicate.

### Morphological characterization and particle size analysis

The shape and surface morphology of the final obtained microspheres were observed by scanning electron microscopy (SEM). The microspheres were added onto double-sided adhesive carbon tape attached to an aluminum stub and then coated with a layer of gold. The coated specimen was photographed by SEM (Hitachi S-4800, Japan) at an acceleration voltage of 5 keV and working distance of 7.4 mm.

The microsphere size and size distribution of the prepared microspheres were measured by a light-scattering particle size analyzer (Ls800, OMEC Ltd., China). Microspheres were dispersed in sodium carboxymethyl cellulose solution (0.5%, w/v) [25] and then analyzed. All measurements were conducted in triplicate.

### In vitro release assay

The *in vitro* release behavior of triptolide loaded microspheres was investigated as follows. Ten milligrams of drug-loaded microspheres were added into a 30 mL cylindrical tube, which contained 25 mL of 0.1 M phosphate buffer solution (PBS, pH 7.4) and sodium azide (0.02%, w/v). These tubes were then incubated in a water bath (37 ± 0.5°C) and shaken horizontally at 72 rpm [26]. At predetermined time intervals, 2 mL of sample solution was withdrawn and replenished with 2 mL of fresh medium maintained at the same temperature. The samples were centrifuged at 5,000 rpm for 10 min, the supernatants were collected for determination, and the precipitate microspheres were put back into the corresponding tubes. The triptolide concentrations were assayed by HPLC as described above. All measurements were conducted in triplicate.

### Release kinetics

To investigate the possible release mechanism of triptolide loaded PLGA microspheres, the *in vitro* release data were fitted by mathematical models such as zero order (Q = kt), first order (Ln(1—Q) = -kt), Higuchi (Q = kt^1/2^) [27] and Korsmeyer-Peppas equation (LnQ = nLnt + k) [28, 29]. In these equations, Q represents cumulative drug release at time t, k is the release rate constant, and n is the exponent indicative of release mechanism. After model fitting and coefficient (R^2^) calculation, the model that gave the highest value of R^2^ was considered to be the most suitable kinetic model for describing the release mechanism.

### Statistical analysis

All data are shown as the mean ± standard deviation (SD) unless otherwise stated. Student’s t test or one-way analysis of variance (ANOVA) was performed to compare the means between groups. A P value less than 0.05 was considered to be significant. Plots of drug release profiles were conducted by Origin 8.6 Software (Origin Lab Corporation, MA, USA). Graphs related to the optimum process were generated using Design-Expert 8.0.5 software (Stat-Easy In., MN, USA).

## Results and discussion

### Experimental design

Drug content can reflect the drug loading efficiency of the microspheres (formulation); encapsulation efficiency can indicate the drug loading efficiency of the preparation process (technique); mean diameter will influence the mobility of microspheres through the needle and the drug release behavior of microspheres; initial release determines the drug onset rate and safe dosage of the prepared microspheres. The above four responses are the key performance indicators in the quality control of microspheres; therefore they were selected to evaluate the prepared microspheres in the experimental design.

As the ratios of max to min for the four responses were less than 3, no transformation was utilized to deal with the response data. For ratios less than 3, the power transforms have little effect.

Polynomial models were selected for the four response (dependent) variables. The suggested fitting mathematical model was automatically selected based on the comparisons of five statistical parameters, including the Lack of Fit p-value, coefficient of determination (R^2^), adjusted multiple correlation coefficient (adjusted R^2^), predicted multiple correlation coefficient (predicted R^2^) and predicted residual sum of squares (PRESS). As shown in Table 4, the "Lack of Fit" p values of the four suggested models implied that the "Lack of Fit" was not significant relative to the pure error. This means that these modes are well-fitted. The R-squared (R^2^) values of the four responses are all greater than 0.95, indicating a good fit or reliability of the models. The "adjusted R^2^" values were the "Pred R-Squared" of 0.9701, which is in reasonable agreement with the "predicted R^2^" values (the differences are less than 0.2) [30], indicating a good correlation between the experimental (actual) and predicted response values. This also implies that the suggested models have good predictability. Moreover, the suggested models have lower PRESS values than the no-suggested models, which indicates the good fitness of the mathematical models.

**Table 4:** Fit summary statistics for response variables in central composite design.

Response	Source	Lack of Fit p-value	R-Squared	Adjusted R-Squared	Predicted R-Squared	PRESS	Remark
Y1	Linear	0.0025	0.8043	0.7676	0.6986	10.80	
	2FI	0.0032	0.8659	0.8041	0.6410	12.87	
	Quadratic	0.5567	0.9911	0.9831	0.9615	1.38	Suggested
	Cubic	0.5246	0.9948	0.9835	0.8952	3.76	Aliased
Y2	Linear	0.0052	0.4904	0.3949	0.2393	1199.32	
	2FI	0.0058	0.6305	0.4600	0.0686	1468.53	
	Quadratic	0.7919	0.9754	0.9532	0.9158	132.75	Suggested
	Cubic	0.9577	0.9831	0.9466	0.9734	41.87	Aliased
Y3	Linear	0.0416	0.8148	0.7800	0.6704	276.08	
	2FI	0.6257	0.9656	0.9497	0.9346	54.76	Suggested
	Quadratic	0.7590	0.9773	0.9568	0.9196	67.39	
	Cubic	0.5058	0.9834	0.9475	0.6384	302.93	Aliased
Y4	Linear	0.0259	0.59433	0.51826	0.295947	144.138	
	2FI	0.0154	0.61145	0.43212	0.277522	147.91	
	Quadratic	0.9422	0.96761	0.93846	0.916865	17.0198	Suggested
	Cubic	0.9812	0.97331	0.91549	0.960884	8.00805	Aliased

Based on the fit summary of Table 4, a quadratic model was suggested for responses Y_1_, Y_2_ and Y_4_, and the 2FI model was suggested for response Y_3_.

"Adeq Precision" measures the signal to noise ratio. A ratio greater than 4 is desirable. The ratios of the four responses (Y_1_: 48.090, Y_2_: 19.225, Y_3_: 29.313 and Y_4_: 25.164) are all greater than 4, which indicates an adequate signal.

**Fig 2:**
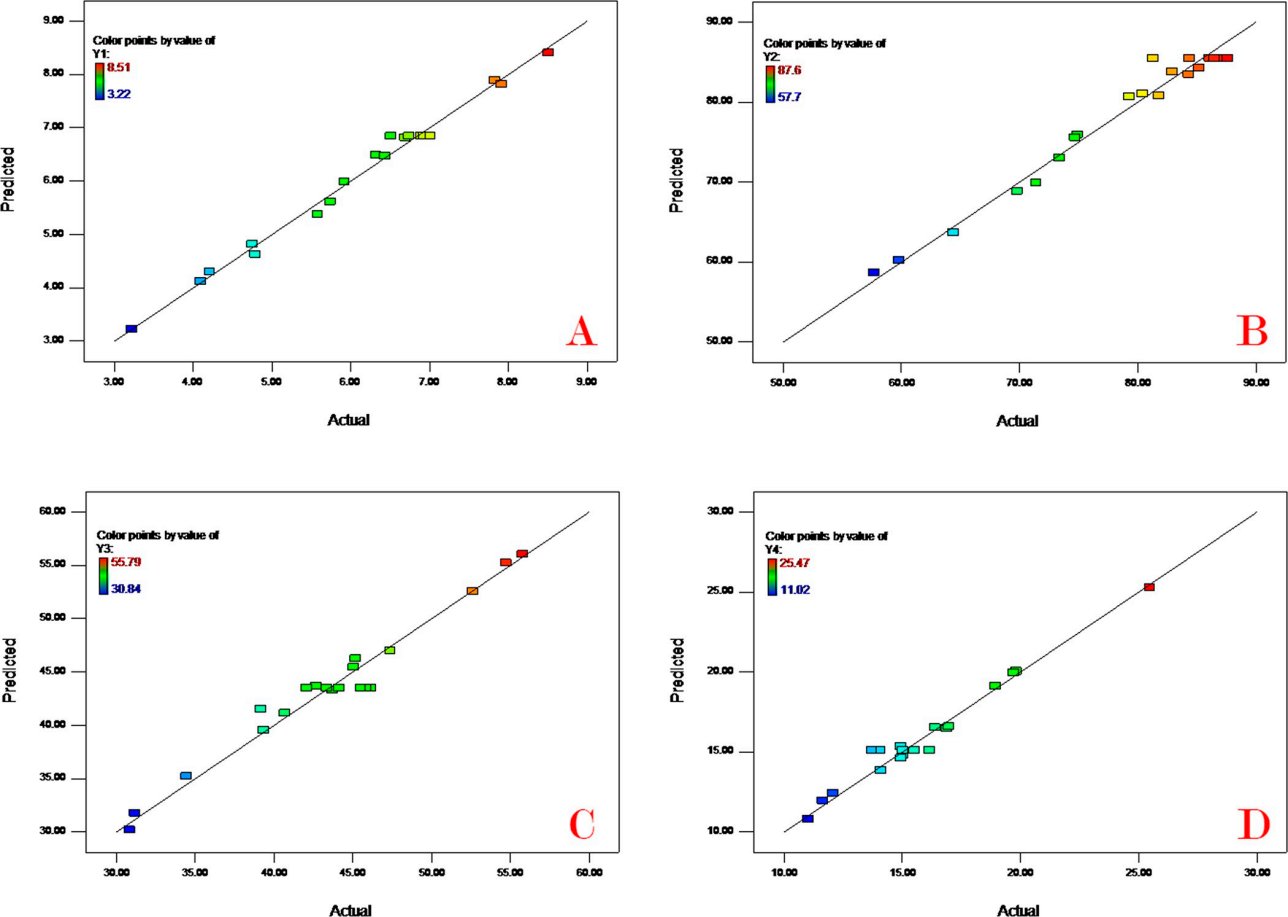
Predicted vs. actual value plots for (A) drug content, (B) encapsulation efficiency, (C) mean diameter, and (D) initial release.

The plots of predicted versus actual values can give us a visual representation on the model satisfactoriness. The predicted versus actual value plots of four responses (Y_1_-Y_4_) are displayed in Fig 2 for drug content, EE, mean diameter and initial release. These plots indicate sufficient agreement between the real data and the data generated by the models.

After meeting the above criteria, these mathematical models suggested by the software were used to plot the relationships between factors and responses and produce visual images for the design space (see the "Response surface and contour plot analysis" section). This is helpful to determine the edge of failure for process parameters beyond which the relevant quality attributes cannot be met.

### Mathematical regression of models

To describe the relationships between the dependent (responses) and independent (factors) variables, mathematical models were generated by the software. The suggested polynomial equations relating the factors and responses in terms of actual factors were obtained and given in Eqs 1–4.

$$Y_{1}=-7.40324+2.89623∙X_{1}+7.51197\times 10^{-3}∙X_{2}+1.15005∙X_{3}+3.69463\times 10^{-3}∙X_{2}∙X_{3}-0.35355∙X_{1}^{2}-7.45492\times 10^{-5}∙X_{2}^{2}-0.082218∙X_{3}^{2}$$ (1)

$$Y_{2}=-10.61230+34.88332∙X_{1}+0.23928∙X_{2}+0.75036∙X_{3}-0.017442∙X_{1}∙X_{2}+0.70711∙X_{1}∙X_{3}+0.035002∙X_{2}∙X_{3}-4.51537∙X_{1}^{2}-9.15958\times 10^{-4}∙X_{2}^{2}-0.81935∙X_{3}^{2}$$ (2)

$$Y_{3}=56.43093-13.28614∙X_{1}+0.19411∙X_{2}+3.33723∙X_{3}-9.28667\times 10^{-3}∙X_{1}∙X_{2}+0.45137∙X_{1}∙X_{3}-0.027047∙X_{2}∙X_{3}$$ (3)

$$Y_{4}=49.62976-20.16373∙X_{1}+0.014908∙X_{2}+0.51124∙X_{3}-1.90919\times 10^{-3}∙X_{1}∙X_{2}-0.066586∙X_{1}∙X_{3}+4.51664\times 10^{-3}∙X_{2}∙X_{3}+2.58135∙X_{1}^{2}-1.82197\times 10^{-4}∙X_{2}^{2}-0.037311∙X_{3}^{2}$$ (4)

where Y_1_, Y_2_, Y_3_ and Y_4_ are actual values for drug content, EE, mean diameter and initial release, respectively. X_1_, X_2_ and X_3_ are actual values for PVA concentration (X_1_), PLGA concentration (X_2_) and theoretical drug content (X_3_), respectively.

The equations represent the quantitative effects of the factors on the responses. The coefficients display how the factors influence the responses. If the coefficient is positive, the response will increase when the factor ranges from low level (-1.732) to a high level (+1.732); the opposite effect will be obtained if the coefficient is negative. According to the magnitude of the coefficients of X_1_ to X_3_ in Eqs 1–4, PVA concentration (X_1_) has the greatest effect (numerical value of coefficient) on responses (Y_1_-Y_4_).

The coefficients of Eq 1 appear as one constant, three linear, three quadratic, and one interaction term. The coefficients imply that for Y_1_ (drug content), the first- and second-order main effects are generated from PVA concentration (X_1_), PLGA concentration (X_2_) and theoretical drug content (X_3_), and the main interaction terms are from X_2_-X_3_.

The coefficients of Eq 2 appear as one constant, three linear, three quadratic, and three interaction terms. The coefficients imply that for Y_2_ (encapsulation efficiency), the first-, second-order and interaction main effects are generated from PVA concentration (X_1_), PLGA concentration (X_2_) and theoretical drug content (X_3_).

The coefficients of Eq 3 appear as one constant, three linear and three interaction terms. The coefficients imply that for Y_3_ (mean diameter), the first-order and interaction main effects are generated from PVA concentration (X_1_), PLGA concentration (X_2_) and theoretical drug content (X_3_).

The coefficients of Eq 4 appear as one constant, three linear, three quadratic, and three interaction terms. The coefficients imply that for Y_4_ (initial release), the first-, second-order and interaction main effects are generated from PVA concentration (X_1_), PLGA concentration (X_2_) and theoretical drug content (X_3_).

### Response surface and contour plot analysis

**Fig 3:**
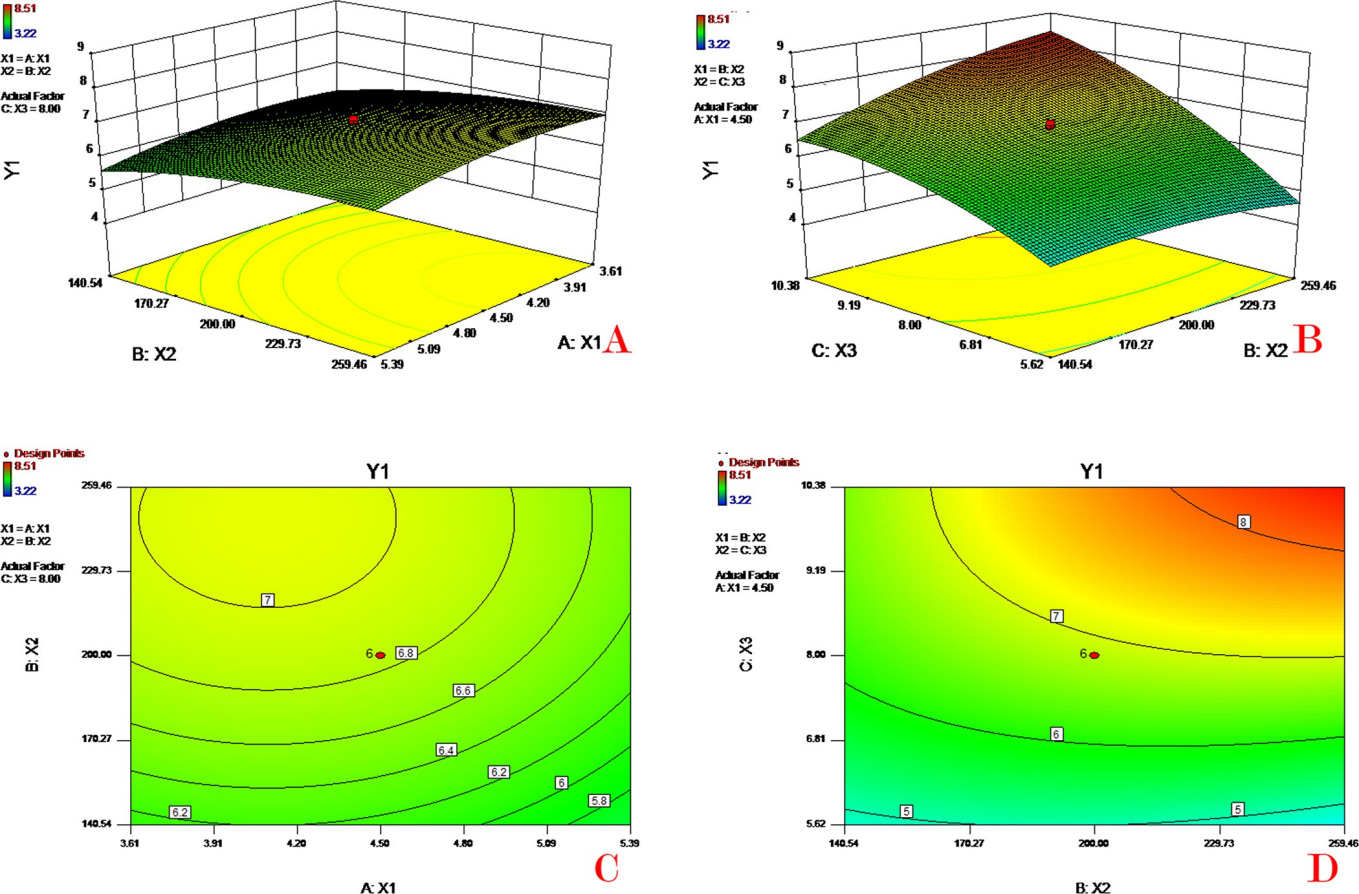
Response surface (A&B) and contour plots (C&D) of PVA concentration (X_1_), PLGA concentration (X_2_) and theoretical drug content (X_3_) on drug content (Y_1_).

**Fig 4:**
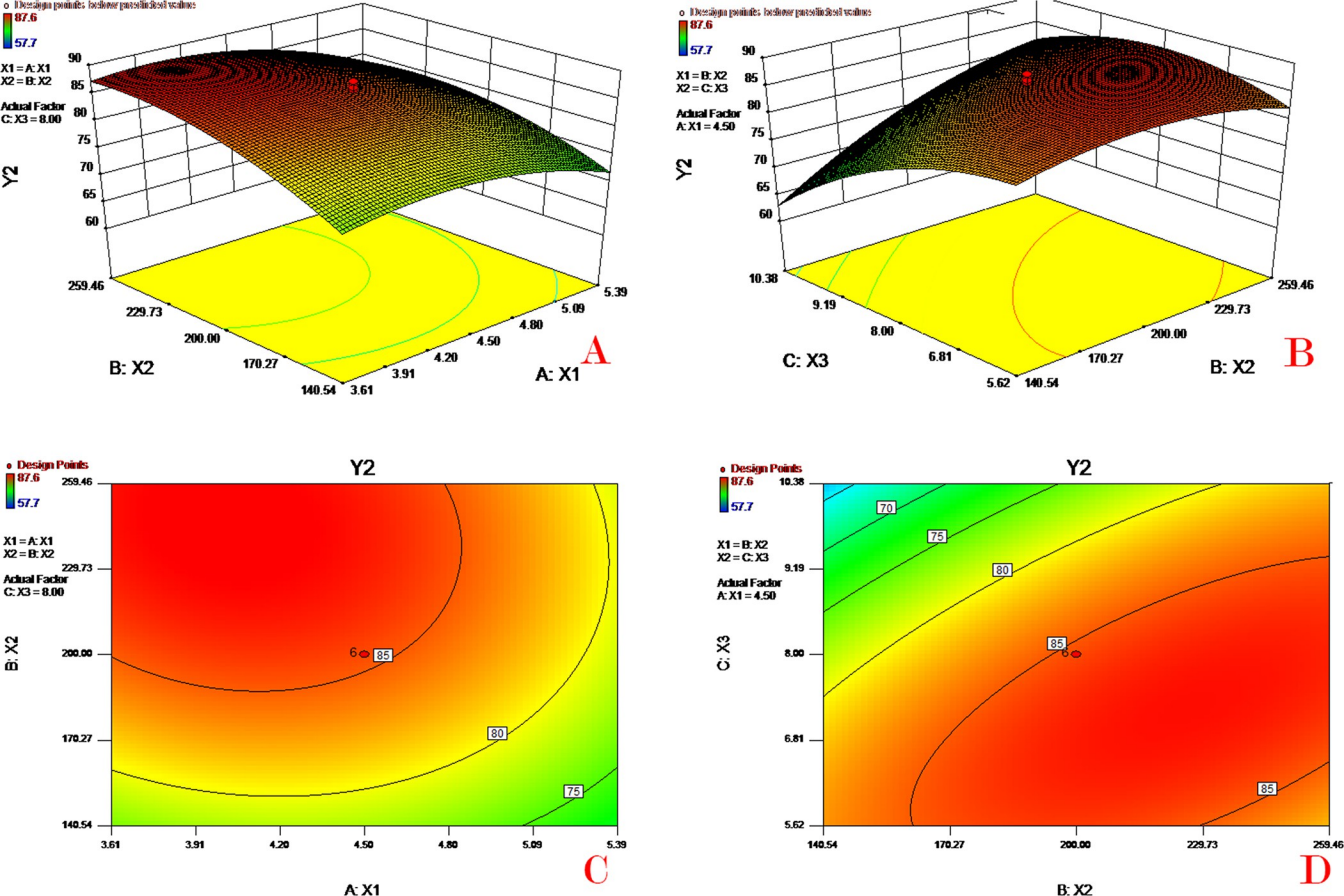
Response surface (A&B) and contour plots (C&D) of PVA concentration (X_1_), PLGA concentration (X_2_) and theoretical drug content (X_3_) on encapsulation efficiency (Y_2_).

**Fig 5:**
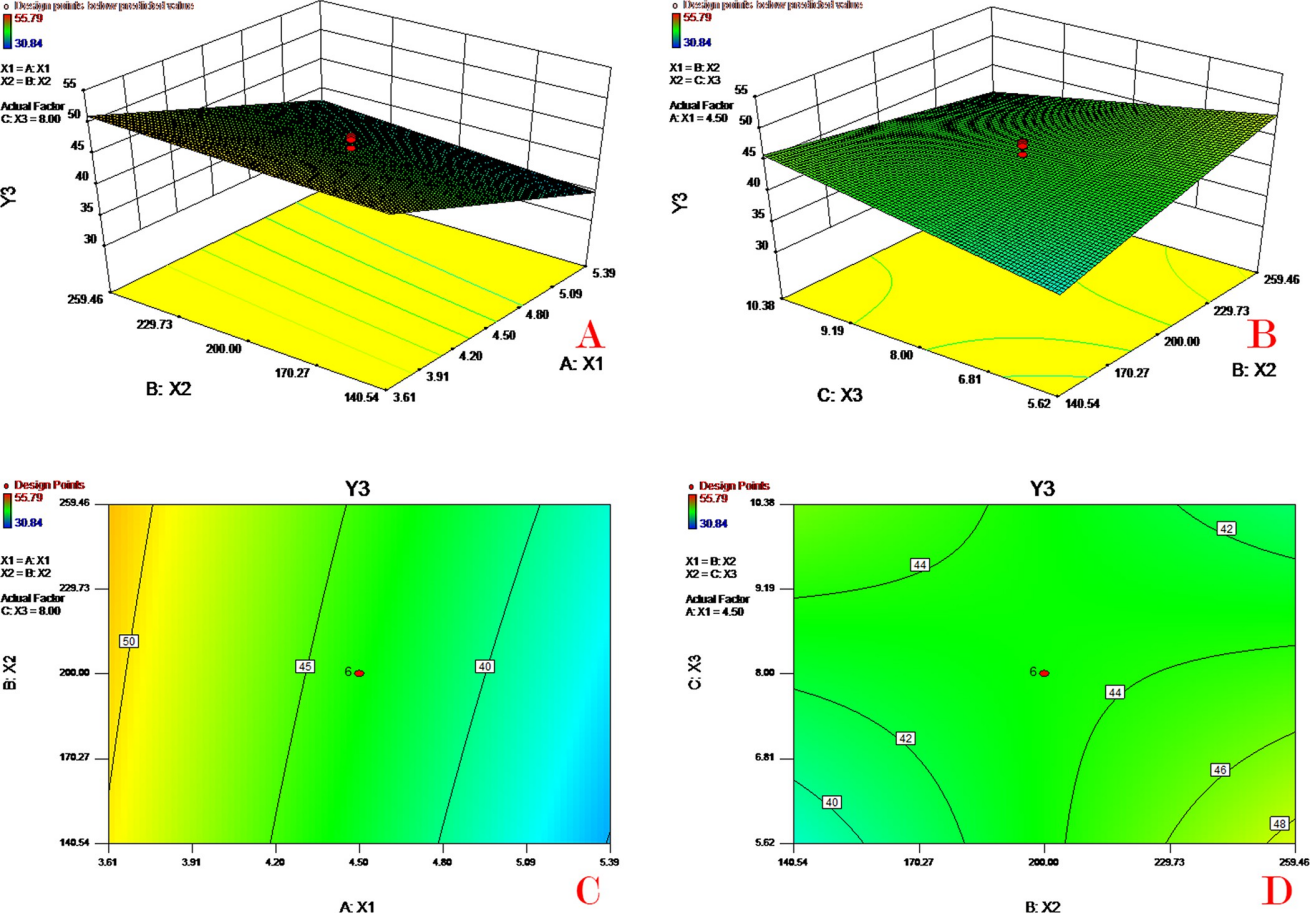
Response surface (A&B) and contour plots (C&D) of PVA concentration (X_1_), PLGA concentration (X_2_) and theoretical drug content (X_3_) on mean diameter (Y_3_).

**Fig 6:**
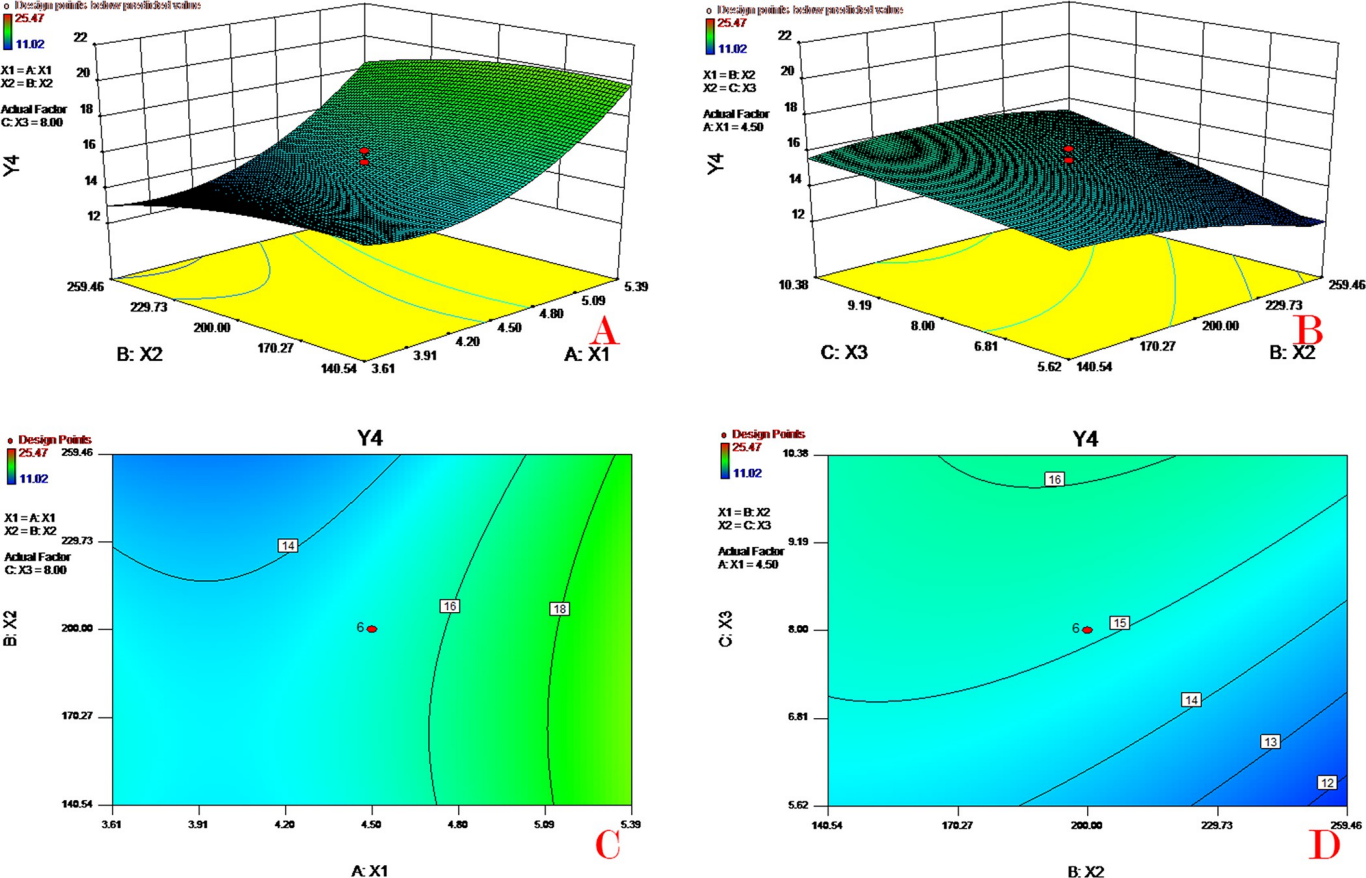
Response surface (A&B) and contour plots (C&D) of PVA concentration (X_1_), PLGA concentration (X_2_) and theoretical drug content (X_3_) on initial release (Y_4_).

The relationships between the dependent and independent variables were demonstrated by the three-dimensional response surface plots and corresponding two-dimensional contour plots generated by the mathematical models (Figs 3–6). These plots are useful for showing the interaction effects of factors on the responses, displaying the effects of two factors on the response at the same time [31].

Fig 3A and 3C depicts the combined effect of PVA concentration (X_1_) and PLGA concentration (X_2_) on drug content (Y_1_). As the PVA concentration increased, the drug content decreased, while the drug content value increased with increasing PLGA concentration. Fig 3B and 3D shows the combined effect of PLGA concentration (X_2_) and theoretical drug content (X_3_) on drug content (Y_1_). These two plots indicate that the drug content value increases slowly as the PLGA concentration or theoretical drug content increases. To avoid the occurrence of high EE with low drug loading, the response of drug content was selected as an important qualifying parameter for the subsequent model optimization. A high PVA concentration in outer water could produce small microspheres with a large surface area resulting in an increase in drug diffusion into the water phase, therefore leading to a low drug content. High PLGA concentrations could slow drug diffusion and thereby increase the drug content (EE).

Fig 4A and 4C depicts the combined effect of PVA concentration (X_1_) and PLGA concentration (X_2_) on encapsulation efficiency (Y_2_). As the PVA concentration increases the value of EE decreases quickly; the EE value increases as the PLGA concentration increases. Fig 4B and 4D shows the combined effect of PLGA concentration (X_2_) and theoretical drug content (X_3_) on encapsulation efficiency (Y_2_). These two plots indicate that the EE value increases dramatically as the PLGA concentration increases or the theoretical drug content decreases. It has been reported that EE is highly affected by many factors, such as the polymer concentration, intrinsic viscosity, diameter of microspheres, and molecular weight of polymers [32]. An increase in the concentration of PVA in the water phase led to a decrease in the EE. A possible reason for this might be that the higher PVA in the outer water phase would increase the drug solubility in the aqueous phase and therefore impair drug EE. Moreover, an increase in PVA would generate a smaller microsphere (diameter) resulting in a lower EE. A higher concentration of PLGA in the oil phase would increase the drug EE, because the enhancement of PLGA concentration would increase the viscosity of the oil phase and hence prevent the drug from diffusing out of the oil phase.

Fig 5A and 5C depicts the combined effect of PVA concentration (X_1_) and PLGA concentration (X_2_) on mean diameter (Y_3_). As the PVA concentration increased, the value of the mean diameter decreased; the mean diameter value increased slightly as the PLGA concentration increased. Fig 5B and 5D shows the combined effect of PLGA concentration (X_2_) and theoretical drug content (X_3_) on mean diameter (Y_3_). Both factors (X_2_, X_3_) have mild effects on the mean diameter in the chosen scope. With the combination of the two factors (X_2_, X_3_), there is a vertex space for the response Y_3._ In such a design space (area) with X_1_ fixed, the mean diameter can obtain a relatively stable value. Moreover, the theoretical drug content has a limited effect on the mean diameter. The increase in PVA concentration could decrease the surface tension of the oil-water phase interface and thus increase the microsphere diameter. The PLGA concentration could influence the viscosity of the oil phase and hence affect the diameter of the microspheres.

Fig 6A and 6C depicts the combined effect of PVA concentration (X_1_) and PLGA concentration (X_2_) on initial release (Y_3_). As the PVA concentration increased, the value of initial release increased dramatically; the initial release value increased slightly as the PLGA concentration decreased. Fig 6B and 6D shows the combined effect of PLGA concentration (X_2_) and theoretical drug content (X_3_) on initial release (Y_4_). The initial release value increases as the theoretical drug content increases or PLGA concentration decreases. According to the above discussion, a high PVA concentration or low PLGA concentration would decrease the microsphere size. The enhancement of the initial release might be due to the smaller diameter and higher surface areas of the microspheres. This is because microspheres with a larger surface/volume ratio have more contact areas for drug diffusion into the release medium.

There are many preparation parameters that may influence the pharmaceutical features of microspheres, such as the homogenization speed, stirring paddle size, and temperature of the preparation process. These factors have a relatively small influence compared with the selected factors (X_1_-X_3_), and they were removed from the CCD to simplify the subsequent formulation optimization.

### Optimization

After the construction of mathematical models, the desirability function approach which can give an assessment of how well the comprehensive goals are satisfied for all responses was provided by the software to conduct the formulation optimization. Such an approach has many advantages, such as being easy to set in the software and flexible to give weight or importance to each response [33]. For these reasons, the desirability function has been widely utilized in the optimization of multiple responses.

For our final goals, we want the microspheres to have high drug content and EE to load more drugs into the preparation and have a small mean diameter and initial release to aid the usage of such drugs. In the numerical optimization process, all goals of the three factors (X_1_-X_3_) were set as "in range", the goals of Y_1_ and Y_2_ were set as "maximize" and the goals of Y_3_ and Y_4_ were set as "minimize", and the other parameters were set by default.

In the numerical optimization process, all goals of the three factors (X_1_-X_3_) were set as "in range", the goals of Y_1_ and Y_2_ were set as "maximize", the goals of Y_3_ and Y_4_ were set as "minimize", and the other parameters were set by default. In the graphical optimization process, all parameters were set automatically by the software. The software provided 8 sets of optimal solutions, and the first recommended solution with a desirability of 0.762 was as follows: 4.48% PVA concentration (X_1_), 259.46 of PLGA concentration (X_2_) and 10.10% theoretical drug content (X_3_). These values predicted 8.32% drug content, 82.87% EE, 41.32 μm mean diameter and 15.11% initial release.

**Fig 7:**
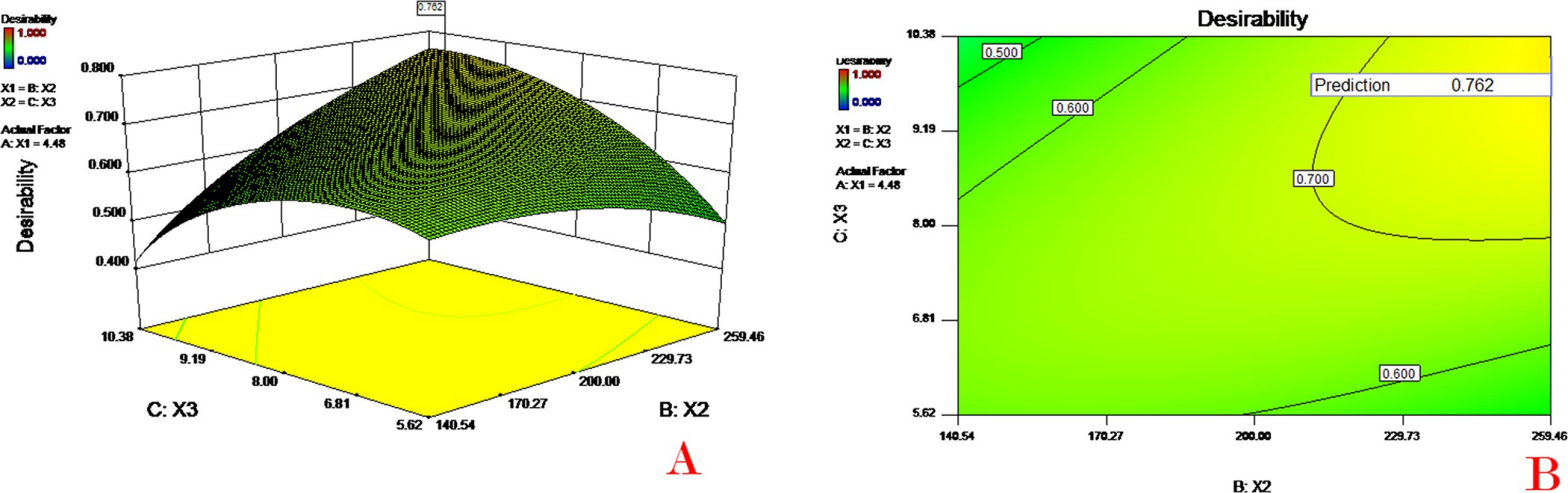
Response surface (A) and contour plots (B) of PLGA concentration (X_2_) and theoretical drug content (X_3_) on the desirability function. At a PVA concentration (X_1_) of 4.48%.

Fig 7A depicts the 3D response surface of the desirability function for the first recommended solution (other solutions were similar to this one). The area of the optimized formulation is shown in Fig 7B, in which the yellow region represents the area satisfying the proposed constraints.

### Confirmation experiments

To confirm the validity of the obtained optimal conditions, confirmation experiments were conducted with the software recommended parameters in triplicate (X_1_ = 4.48%, X_2_ = 259.46 mg/ml, X_3_ = 10.10%). Table 5 represents the predicted values and experimental results for the four response variables at the optimal levels of the three factors. The errors between the observed (measured) and predicted values of the four responses were less than 5%, showing the good predictability and reliability of the constructed models [34].

**Table 5:** Predicted and measured response values at the optimal levels of factors.

Response variables	Predicted value	Observed value	%Error^a^
Drug content	8.32	7.96	-4.52
Encapsulation efficiency	82.87	80.16	-3.38
Mean diameter	41.32	42.36	2.45
Initial release	15.11	14.48	-4.35

^a^Error (%) = (Observed value—Predicted value) / Observed value×100%

### Morphology, particle size and drug loading

**Fig 8:**
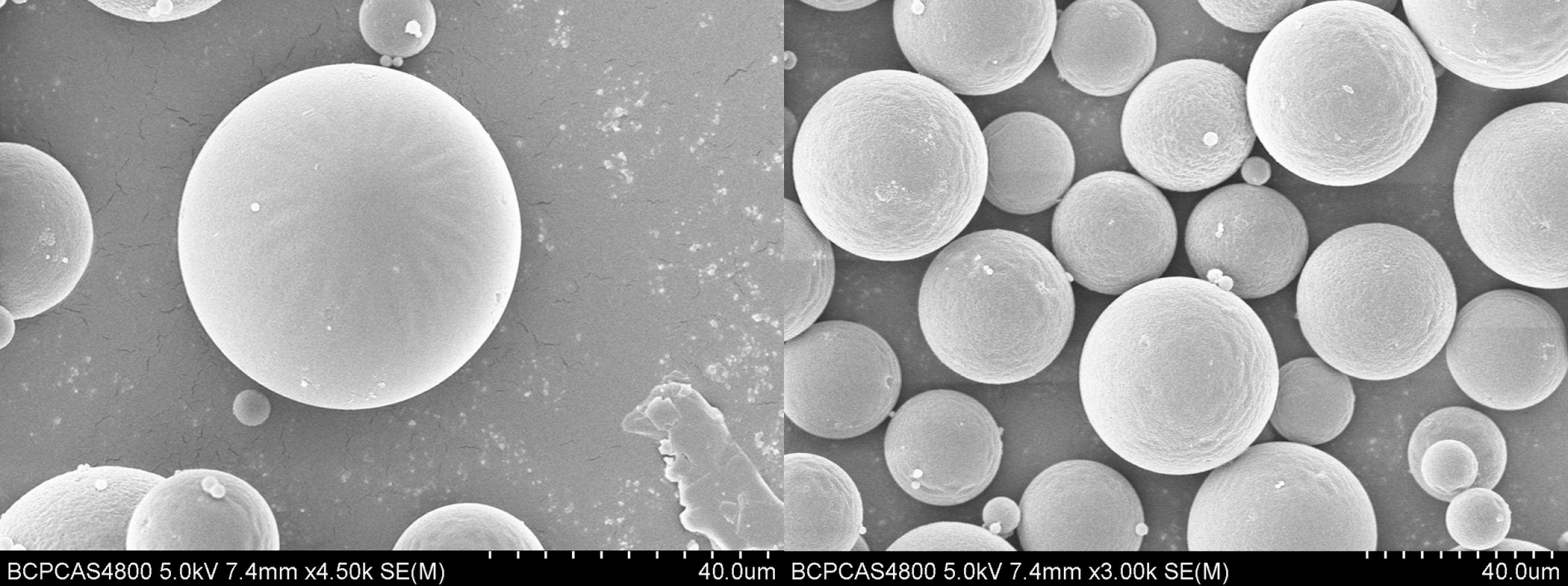
Micrograph of triptolide loaded PLGA microspheres by scanning electron microscopy.

The surface morphology of microspheres prepared with the optimized formulation was observed by SEM. The prepared microspheres were spherical and smooth without any pores on their surface (Fig 8). Moreover, the microspheres appear to be regular and homogeneous.

**Fig 9:**
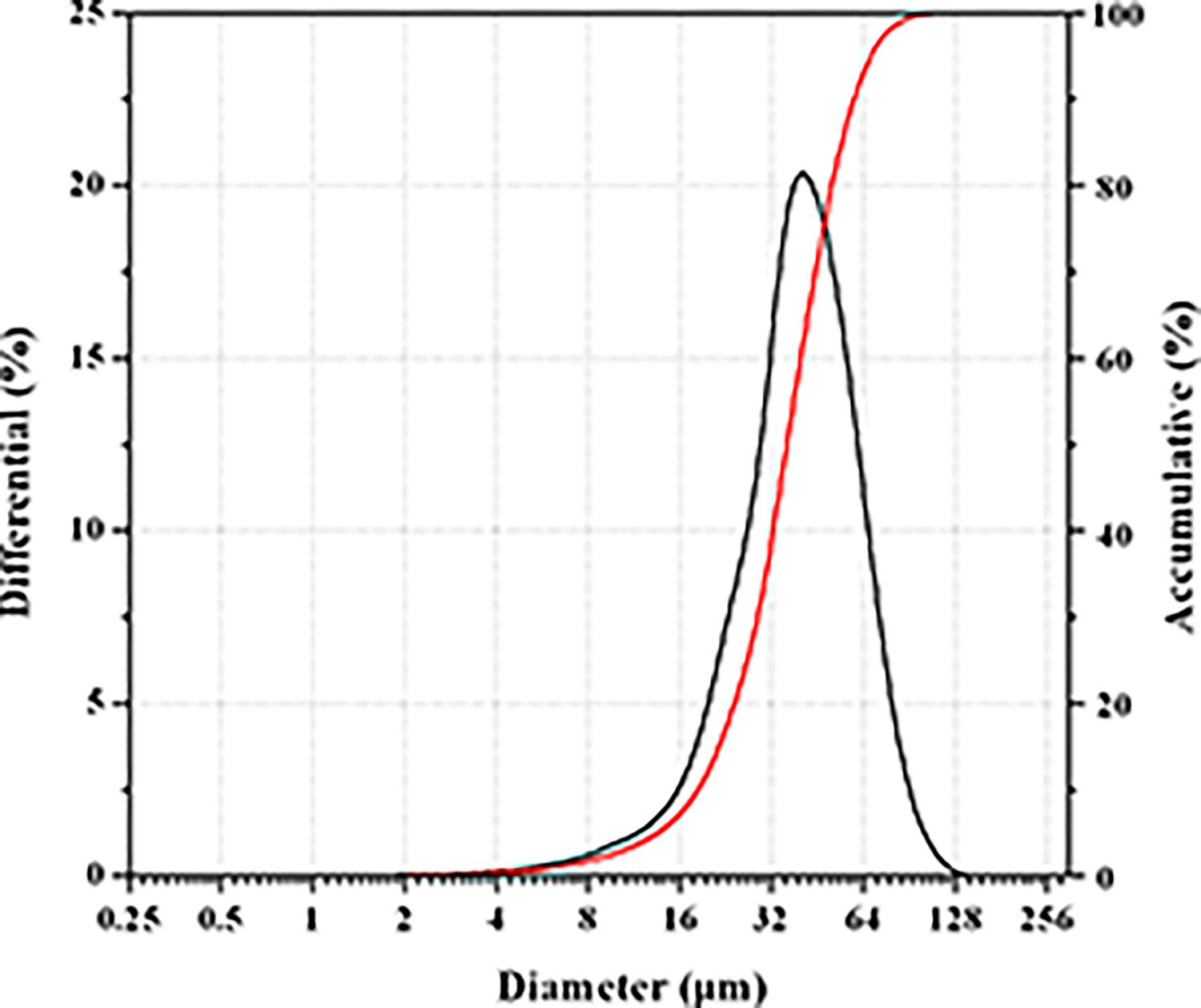
Particle size distribution of triptolide loaded PLGA microspheres.

Particle size is an important feature of microspheres, as it can influence the drug content, drug EE, drug release rate, and initial burst release of microspheres [32]. As shown in Fig 9, the mean diameter of the microspheres was approximately 40 μm, with an average value of 42.36 ± 1.33 μm. The particle size of the microspheres is uniform with a small span of size distribution (1.03 ± 0.05). These results are consistent with the above SEM results.

After the drug content assay, the drug content and EE of the prepared microspheres were calculated as 7.96 ± 0.19% and 80.16 ± 3.36, respectively.

The preparation method used in this work is emulsion solvent evaporation, which was adopted with some modifications reported by Wu et al. [11]. Since this technique requires no special equipment [35] and only mild conditions such as constant stirring and ambient temperature [36], it is widely applied in the preparation of microspheres and is superior to other methods.

### In vitro drug release

**Fig 10:**
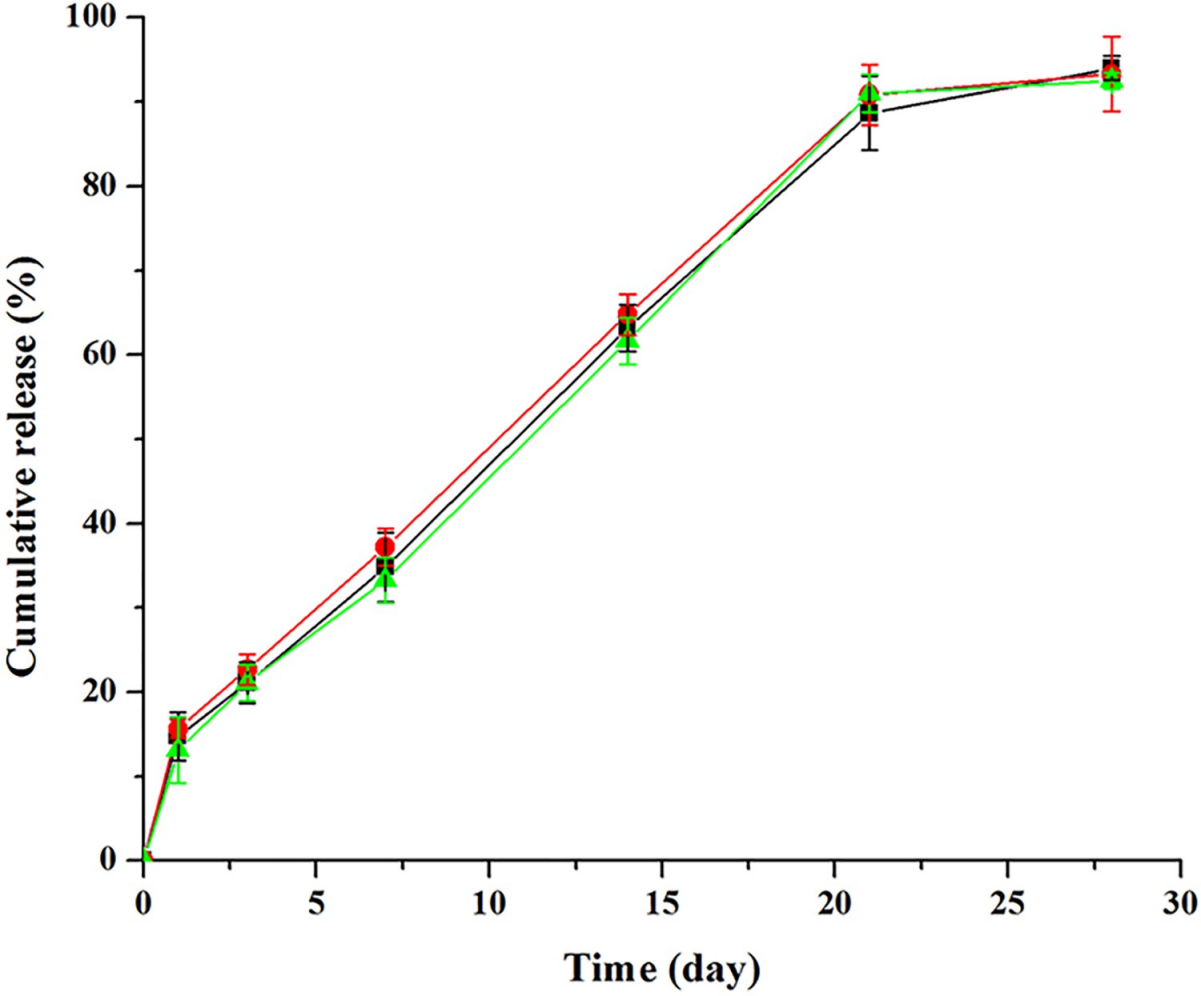
*In vitro* cumulative drug release from triptolide loaded PLGA microspheres in PBS pH 7.4 at 37°C (mean± SD, n = 3).

Fig 10 displays the *in vitro* release profiles of three batches of triptolide loaded microspheres. The *in vitro* drug release behaviors of the three batches showed good consistency. After an initial burst release of approximately 14% within the first 24 h, the triptolide release rate slowed down and remained constant from Day 2 to Day 21 (3.5% released per day); after that, the release rate slowed down gradually and reached a plateau phase (after Day 21); over 90% of the drug was released by Day 28. In short, the triptolide release feature from the prepared microspheres can be attributed to the interactions between the drug and the polymer.

Initial burst release is a great challenge for microspheres. Approximately 10%-80% of the total drug loading will be released from microspheres within the first day in the burst release period [37]. This dose dumping may pose a serious toxicity risk for the users of microspheres and is a major obstacle to the development of microsphere products. To prepare microspheres with low burst release, numerous strategies have been developed, for example using different matrices, modifying the encapsulated drugs and employing special preparation methods [38, 39]. For the narrow therapeutic index drug triptolide, the issue of reducing the abrupt release becomes more prominent. Considering that the designed drug release period is 30 days, the initial burst release of triptolide microspheres prepared (~14%) in this paper should at least be reduced to 3% (1/30) to achieve a safe and optimal therapeutic level and decrease side effects. Now, the important issue facing treptolide is not the absence of sustained-release (SR) properties but rather its narrow therapeutic window. This calls for research on development of new emulsion preparation methods, pre-release of initial burst release and / or new materials to overcome this outstanding issue. *In vitro* drug release tests are commonly employed to assess the initial burst release of microsphere products and thus ensure clinical safety.

As reported in the literature, commercial preparations of TWHF can cause severe drug reactions such as reproductive toxicity, hepatotoxicity and renal cytotoxicity. Studies have also demonstrated that triptolide-induced toxicity depends on dosage and use time [7]. It can be inferred that the control of the amount of initial release and regular medical examinations of the users are prerequisites for the clinical usage of triptolide SR microspheres. If triptolide SR microspheres exhibit an accidental burst upon administration, they may cause the severe reactions described above, or even organ failure (liver and /or kidney). In such cases, emergency surgery is required to remove the implanted microspheres. This suggests that the control of burst release is critical for the quality of triptolide microspheres. Moreover, the potential clinical benefits of triptolide SR microspheres may not be justified unless the *in vitro* drug release characterization is substantiated by *in vivo* studies.

### Release kinetics

These *in vitro* release data were fitted with zero-order (F_t_ = 3.375t + 9.718, R^2^ = 0.957), first-order (Ln(100—Ft) = -0.100t + 4.684, R^2^ = 0.965), Higuchi (Q = 18.94t1/2–6.009, R^2^ = 0.971) and Korsmeyer-Peppas (Ln(Q) = 0.600Ln(t) + 2.540, R^2^ = 0.975) equations. The data best fit the Korsmeyer-Peppas model with the highest R^2^ value, indicating that the *in vitro* triptolide release was mainly controlled by more than one type of diffusion release mechanism (Fickian and non-Fickian diffusion) [40].

## Conclusions

In this study, triptolide loaded PLGA microspheres were successfully prepared by an O/W emulsion solvent evaporation method. The formulation of triptolide microspheres was developed and optimized successfully utilizing RSM coupled with CCD. A series of experiments given by the central composite design were conducted and the related data were processed by the software, which provided the optimized factor levels. The predicted optimal conditions were further confirmed by the validated experiments and showed good consistency with the predicted values of the responses. The optimized microspheres displayed a sustained release of triptolide *in vitro* over 4 weeks, which was well fitted with a Korsmeyer-Peppas model. However, all the evaluations in this paper were *in vitro*, and further *in vivo* experiments are being performed. The initial burst release issue (reduced from ~14% to 3%) should also be specifically investigated in future studies. *In vivo* studies are required to validate the *in vitro* drug release properties of triptolide microspheres and thus justify their potential clinical benefits. The extended release feature of triptolide from such microspheres could provide a reliable and convenient alternative option for the long-term treatment of RA. Once a new formulation is developed with an initial burst release reduced to 3% or less, and the in *vitro* release data are substantiated with *in vivo* studies, this triptolide-loaded PLGA microsphere formulation may only need to be injected once a month, greatly reducing the burden for RA patients to take medication daily.

## Supporting information

S1 File Research data of the paper. (XLSX)
